# Investigating the crowding effect on letters and symbols in deaf adults

**DOI:** 10.1038/s41598-024-66832-1

**Published:** 2024-07-12

**Authors:** Veena Kamble, Margot Buyle, Virginie Crollen

**Affiliations:** Institut de Recherche en Sciences Psychologiques, Université Catholique de Louvain, Place de l’Université, Louvain-la-Neuve, Belgium

**Keywords:** Deaf, Visual crowding, Letter identification, Reading, Periphery, Human behaviour, Visual system

## Abstract

Reading requires the transformation of a complex array of visual features into sounds and meaning. For deaf signers who experience changes in visual attention and have little or no access to the sounds of the language they read, understanding the visual constraints underlying reading is crucial. This study aims to explore a fundamental aspect of visual perception intertwined with reading: the crowding effect. This effect manifests as the struggle to distinguish a target letter when surrounded by flanker letters. Through a two-alternative forced choice task, we assessed the recognition of letters and symbols presented in isolation or flanked by two or four characters, positioned either to the left or right of fixation. Our findings reveal that while deaf individuals exhibit higher accuracy in processing letters compared to symbols, their performance falls short of that of their hearing counterparts. Interestingly, despite their proficiency with letters, deaf individuals didn’t demonstrate quicker letter identification, particularly in the most challenging scenario where letters were flanked by four characters. These outcomes imply the development of a specialized letter processing system among deaf individuals, albeit one that may subtly diverge from that of their hearing counterparts.

## Introduction

Reading is a highly advanced skill that is usually acquired early in life. It depends not only on language skills, such as phonological and orthographic decoding, but also on various visual processes. Skilled reading in adults indeed relies on a series of saccadic eye movements. These saccades are interspersed with brief pauses known as fixations, during which the eyes remain relatively stationary and gather visual information^1,2^. With the recent advances in eye tracking technology, it has been determined that the size of the visual span from which information is obtained during a fixation extends from 3 to 4 letters to the left and up to 15 letters to the right of a fixation position^3^. Letters’ visibility within this span is importantly influenced by 3 visual factors: acuity (i.e., the closer a letter is to fixation, the more visible it is), spatial attention (i.e., the closer a letter is to the focus of spatial attention, the more visible it is) and crowding^4,5^.

Crowding is a perceptual phenomenon that refers to the impairment of identifying a target stimulus due to the presence of nearby stimuli or flankers. It occurs in the foveal area but is more pronounced in the periphery, increasing at a faster rate with eccentricity^6,7^. A typical task used to measure the crowding effect on letter recognition is the flanker task^8^. In this task, a target letter (e.g., “T”) and several distractor letters (e.g., “H”, “K”, “X”, etc.) are presented briefly (to avoid speed-accuracy trade-off). The task for the participant is to identify or recognize the target letter while ignoring the distractors. The distance between the target letter and the distractors can vary, allowing researchers to examine how crowding affects letter recognition at different spatial scales^9,10^. When flanking letters are close to the target, excessive feature integration makes the identification of the target letter difficult^11,12^. However, after extensive exposure to written language, letters engage in a specialized neural mechanism reducing the crowding effects compared to other types of objects. Becoming a skilled reader therefore involves the emergence of a specialized system prioritizing letter recognition over other characters, such as symbols^13–15^.

As reading engages both language and visual processes, its development could follow a unique trajectory in deaf signers who not only have little or no access to the sounds of the language they read^16^ but also experience changes in the distribution of visual attention (for reviews, see^17,18^). Accordingly, with this idea, deaf individuals were shown to perceive and attend to more letters within a single fixation than their hearing counterparts, ranging from 10 letters to the left^19^ to 18 letters to the right of fixation^20^. Deaf readers are also more sensitive to string length than their hearing peers^21^. They exhibit, while spelling, confusions of letters with similar visual characteristics (e.g., letters with similar height such as t and d; letters with descenders such as p and g)^22^. They demonstrate, unlike their hearing counterparts, a preference for matched-case (EDGE-EDGE) over mismatched-case identity primes (edge-EDGE) in behavioral^23^ as well as in EEG priming tasks^24^. They finally demonstrate, in a lexical decision task, smaller ERPs amplitudes for pseudowords which had a consistent outline shape (e.g., mofor) to those with an inconsistent outline shape (e.g., mosor) relative to their base word (motor)^25^; see also^26^ for similar results).

If these studies all suggest that deaf readers probably present a higher sensitivity to the visual properties of words (see also^27–30^ for similar conclusions) than readers with normal hearing, they all limited their research focus to the word recognition level. Studies examining the nature of letter representation per se are, in contrast, particularly scarce. The few existing studies moreover reached inconsistent conclusions, some suggesting that letter recognition is not impacted by deafness^31^, others highlighting letter identification difficulties in some orthographies (Arabic and Turkish) but not in others (Hebrew, English and German)^32^. As letters are the building blocks of words^33^, it is crucial to further examine the impact deafness may have at this level of the reading circuitry.

The present study will examine letter identity coding in deaf signers by exploring the crowding effect using a flanker task. Deaf and hearing adults will be shown letters and symbols, either in isolation or within a string of three or five characters, to the left or right of fixation. Participants will then perform a two-alternative forced choice (2AFC) task to determine which character out of two was presented in the middle of the string. If deafness does not affect the typical crowding effect, letter identification should be better than symbol identification, and crowding should increase with the number of flankers. However, since deaf individuals experience changes in visual attention distribution, deafness may ultimately shape crowding, either facilitating or hindering the identification of letters and symbols.

## Methods

### Participants

The study population consisted of twenty-one deaf adults (9 males, 3 left-handed, M_age_ = 39.3 ± 13 years), and twenty-one hearing controls adults (HC) (8 males, 1 left-handed, M_age_ = 36.10 ± 14.37 years) (see Table 1 for a detailed description of the deaf and HC participants) who received compensation of 10 euros for their participation. Hearing participants were Dutch or French native speakers, and deaf participants communicated in VGT (Vlaamse Gebarentaal: sign language used in the Dutch part of Belgium) or in LSFB (Langue des Signes de Belgique Francophone: sign language used in the French part of Belgium). None of the deaf participants were diagnosed with additional disabilities, 19 reported congenital deafness, 1 reported prelingual deafness (at 1 year of age), and 1 reported post-lingual deafness (at 3 years of age). Hearing participants were matched with deaf participants in terms of age, [*t* (*40*) = *0.78*, *p* > *0.3*], educational level, [*t* (*40*) = *− 1.68*, *p* > *0.05*], gender, [*X*^*2*^ (*1*, *42*) = *0.000*, *p* = *1*], handedness, [*X*^*2*^ (*1*, *42*) = *0.22*, *p* > *0.3*], and mother tongue (French vs. Dutch), [*X*^*2*^ (*1*, *42*) = *0.000*, *p* = 1]. All participants reported having normal or corrected to normal vision. Written informed consents were signed by all participants. The study was approved by the “Comité d’Ethique hospitalo-facultaire Saint-Luc-UCLouvain” (2019/13JUI/256) and the procedures were in line with the Declaration of Helsinki.

**Table 1:** Characteristics of participants.

Sub	Age	Sex	Language	Handedness	Formal school years (after primary school)	Deafness onset	Etiology	Deafness level
1	56	F	Dutch	R	13	Birth	Hereditary	Profound 3rd degree
2	48	M	Dutch	L	6	Birth	Rubella	Complete
3	23	F	Dutch	R	6	Birth	Genetic	Profound
4	26	F	Dutch	R	7	1 y	Unknown	Profound
5	26	F	Dutch	R	12	3 y	Meningitis	Severe
6	48	M	Dutch	R	6	Birth	O_2_ insufficiency	Profound
7	51	M	Dutch	R	15	Birth	Meningitis	Profound
8	28	F	Dutch	R	14	Birth	Genetic	Severe and profound
9	50	M	Dutch	L	7	Birth	Genetic	Profound
10	37	F	Dutch	R	12	Birth	Genetic	Profound
11	49	F	Dutch	R	7	Birth	Rubella	Profound
12	23	F	French	R	9	Birth	Unknown	Complete
13	43	M	French	R	5	Birth	Hereditary	Profound
14	24	F	French	R	11	Birth	Unknown	Severe left ear/profound right ear
15	20	M	French	R	7	Birth	Unknown	Severe left ear
16	53	M	French	R	6	Birth	Hereditary	Complete
17	53	F	French	R	6	Birth	Hereditary	Complete
18	35	M	French	R	3	Birth	Unknown	Profound
19	63	F	French	R	9	Birth	Hereditary	Complete
20	35	M	French	L	8	Birth	Genetic	Profound
21	37	F	French	R	12	Birth	Nerve atrophy	Profound/severe
22	55	F	Dutch	R	9	–	–	
23	23	M	Dutch	R	12	–	–	
24	23	M	French	R	9	–	–	
25	22	F	French	R	10	–	–	
26	24	F	French	R	15	–	–	
27	20	F	French	R	8	–	–	
28	32	F	French	R	15	–	–	
29	50	M	Dutch	R	11	–	–	
30	57	F	French	R	6	–	–	
31	49	M	Dutch	R	6	–	–	
32	66	M	Dutch	R	11	–	–	
33	31	M	Dutch	R	16	–	–	
34	21	F	French	R	10	–	–	
35	51	F	Dutch	R	9	–	–	
36	39	M	Dutch	R	8	–	–	
37	38	F	Dutch	R	9	–	–	
38	25	F	French	R	14	–	–	
39	36	F	French	L	7	–	–	
40	49	M	French	R	7	–	–	
41	20	F	Dutch	R	9	–	–	
42	27	F	French	R	15	–	–	

*R* Right-handed; *L* Left-handed; *F* Female; *M* Male; *y* Years.

### Procedure

**Figure 1:**
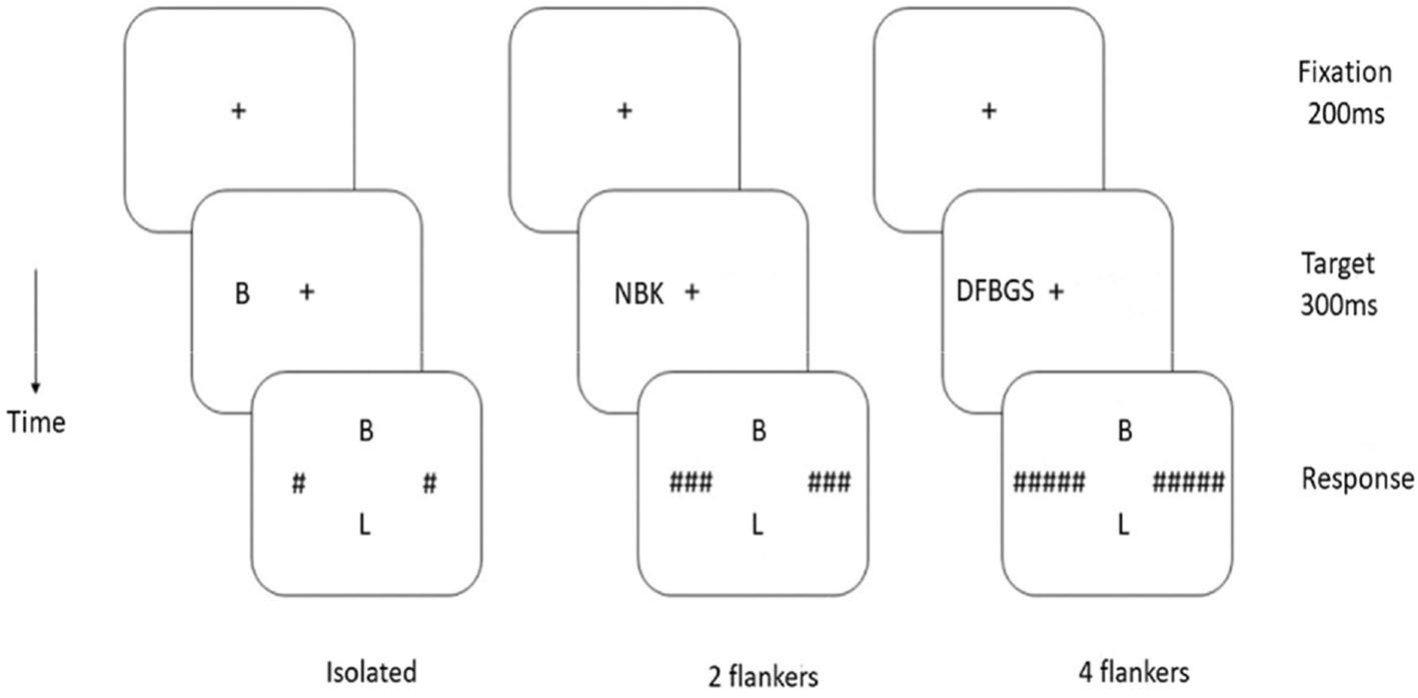
Illustration of the experimental design. The experiment started with a 200 ms display of a fixation cross, succeeded by a 300 ms presentation of the stimulus, comprising either a letter or a symbol surrounded (or not) by flankers. Subsequently, a post-mask was shown, alongside the simultaneous presentation of the two response options. In this instance, the target stimulus was the letter “B,” consistently presented in the left visual field (LVF) across all conditions.

The task used in this study requires participants to identify the middle character of a character string presented briefly to the left or right of fixation. Each trial began with the presentation of a fixation cross at the center of the screen for 200 ms and was followed by the presentation of a character string for 300 ms either on the left or right side of fixation. A backward mask comprising hash marks then replaced the characters (with the number of hash marks matching the number of characters in the string), while simultaneously, two characters were displayed, one above and one below a vacant space at the center of the screen. Participants had to decide which of these two characters had been presented before (two alternative forced choices, 2 AFC). They were asked to respond as quickly as possible by pressing one of two response keys while the mask remained on the screen until the participant responded (see Fig. 1).

### Stimuli and design

Nine uppercase graphemes corresponding to the following consonants (B, D, F, G, K, N, L, S, T) and nine symbols ((%, /, ?, @,}, < , £, §, μ) were used [adapted from^14^]. Each target stimulus (*n* = 18) was presented twice at each position (left vs. right of fixation) and in each condition (isolated, flanked with 2 characters, flanked with 4 characters), leading to a total of 216 trials. These 216 trials were divided into six blocks of 36 trials. Each stimulus was presented 12 times as a flanker and 12 times as an incorrect alternative. The incorrect alternative presented for the forced choice was never the flanking character of the target. The flankers always belonged to the same category of characters (letters versus symbols) as the target.

The task was created using the E-Prime software (Psychology Software Tools, Pittsburgh, PA, USA). Participants were seated approximately 60 cm away from the computer screen. Stimuli were presented in white on a black background in Courier New font size 18. Each letter sustained a visual angle of 1°. Stimuli were presented on each side of the fixation cross at an eccentricity ranging from 3 to 7 degrees of visual angle, a range corresponding to the size of the deaf perceptual span. Participants first viewed the instructions of the task. They then practiced 20 trials to familiarize themselves with the task and subsequently performed the six experimental blocks. The entire task lasted approximately 15 min. Instructions in Dutch and French were given in both oral and written form, as well as instructions in sign language, for deaf participants.

## Results

Analyses were performed using R Statistical Software (version 4.3.0; R Core Team 2021)^34^. Accuracy and reaction time (RT) were treated as dependent variables in the model. A 2 × 3 × 2 × 2 factorial design was carried out on both measures, with Character (2 levels: letter and symbol), Condition (3 levels: isolated, 2 flankers, and 4 flankers), Side (2 levels: left and right), and Group (2 levels: Deaf and Hearing Controls (HC)) as factors. All possible interactions between the predictors were tested. Fixed terms were considered significant when their inclusion led to a noticeable improvement in goodness-of-fit, as indicated by the likelihood ratio tests. Statistical significance was established at *p* < 0.05 for all computations. Accuracy was analysed with generalized linear mixed models using the glmmTMB package (version 1.1.7)^35^ Reaction times for correct responses were analysed with linear mixed effects regression models using the lmertest package (versions 3.1–3)^36^. As the reaction times data follow normal distribution, no transformations were made. To avoid over-parametrisation, random slopes were not included in the model^37^. In case of follow-up, pairwise comparisons were conducted with the emmeans package^38^, and a Bonferroni correction of the p-values was implemented to control for multiple comparisons. Estimates, standard errors (SE), and z (accuracy) or t (reaction time) values are reported, along with p-values.

### Accuracy

**Figure 2:**
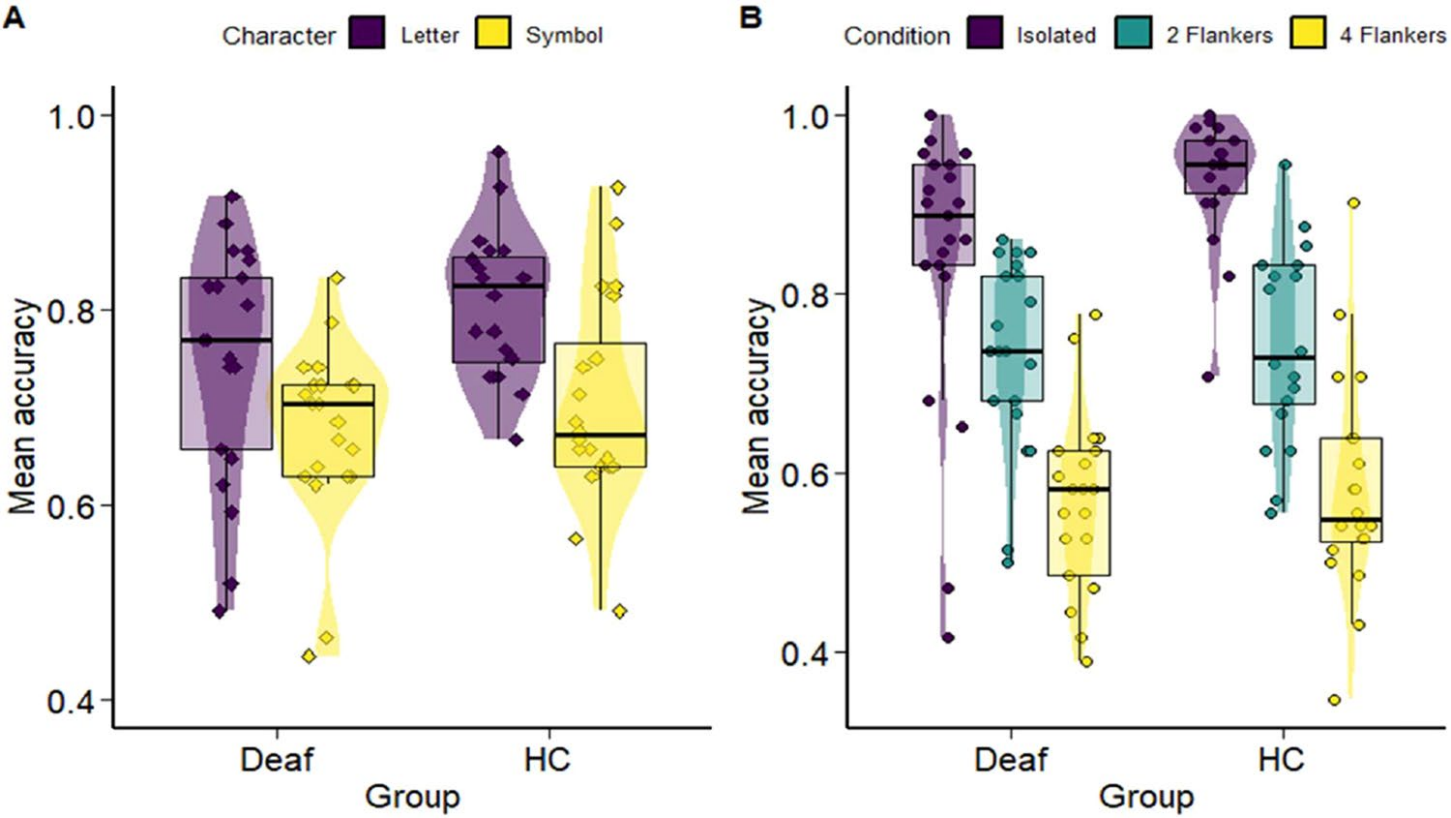
Accuracy results. (**A**) Violin plot depicting the overall accuracy performances of deaf and hearing controls (HC) in the letter (purple) and symbol (yellow) identification tasks. (**B**) Violin plot depicting the accuracy performances of deaf and hearing controls (HC) in isolated (purple), 2 flankers (green), and 4 flankers (yellow) conditions.

A generalized linear mixed model was fitted to predict accuracy with character, condition, side, and group (formula: Accuracy ~ character * condition * side * group). The model included subjects as random effects (formula: ~ 1 | subject) and highlighted a main effect of character, *χ*^*2*^(*1*) = *107.94*, *p* < *0.001*. Letters’ identification was indeed more accurate than symbols’ identification (*estimate* = *0.58*, *SE* = *0.05*, *z* = *10.21*, *p* < *0.001*). The condition effect was also significant, *χ*^*2*^(*2*) = *860.31*, *p* < *0.001*. Accuracy was higher in the isolated condition compared to the condition with 2 flankers (*estimate* = *1.14*, *SE* = *0.07*, *z* = *14.44*, *p* < *0.001*). The condition with 2 flankers was better performed than the condition with 4 flankers (*estimate* = *0.83*, *SE* = *0.05*, *z* = *14.29*, *p* < *0.001*). The group effect was also significant, *χ*^*2*^(*1*) = *5.71*, *p* < *0.05*, with the deaf being less accurate than the hearing controls (*estimate* = *− 0.43*, *SE* = *0.17*, *z* = *− 2.46*, *p* < *0.05*). The side effect did not reach significance, *χ*^*2*^(*1*) = *0.87*, *p* > *0.3*. Results demonstrated a character x group interaction, *χ*^*2*^(*1*) = *6.63*, *p* < *0.05*. Although both groups processed letters more effectively than symbols (*estimate* *for* *the* *deaf* *group* = *0.44*, *SE* = *0.07*, *z* = *6.19*, *p* < *0.01*; *estimate* *for* *the* *HC* *group* = *0.71*, *SE* = *0.08*, *z* = *8.63*, *p* < *0.01*) (see Fig. 2A), subsequent pairwise comparisons revealed that the accuracy in letter processing was lower in the deaf group compared to the hearing control group (*estimate* = *-0.57*, *SE* = *0.18*, *z* = *− 3.05*, *p* < *0.01*). On the contrary, there were no significant group differences in the processing of symbols (*estimate* = *− 0.30*, *SE* = *0.18*, *z* = *− 1.65*, *p* > *0.1*). The analysis finally showed a significant condition × group interaction, *χ*^*2*^(*2*) = *49.13*, *p* < *0.01*. The effects of crowding was evident in each group (deaf: isolated vs 2 flankers *estimate* = *0.68*, *SE* = *0.09*, *z* = *7.21*, *p* < *0.01;* 2 flankers vs 4 flankers *estimate* = *0.80*, *SE* = *0.08*, *z* = *9.91*, *p* < *0.01*; HC : isolated vs 2 flankers *estimate* = *1.60*, *SE* = *0.12*, *z* = *12.88*, *p* < *0.01;* 2 flankers vs 4 flankers *estimate* = *0.86*, *SE* = *0.08*, *z* = *10.33*, *p* < *0.01*) (see Fig. 2B). Pairwise comparisons revealed that the accuracy of the isolated condition was lower in the deaf group than in the HC group (*estimate* = *− 1.07*, *SE* = *0.21*, *z* = *− 5.06*, *p* < *0.01*). There was, in contrast, no group difference in the 2 (*estimate* = *− 0.15*, *SE* = *0.18*, *z* = *− 0.79*, *p* = *1*) and 4 flankers conditions (*estimate* = *− 0.09*, *SE* = *0.18*, *z* = *− 0.49*, *p* > *0.1*). No other effects were significant.

### Reaction times

**Figure 3:**
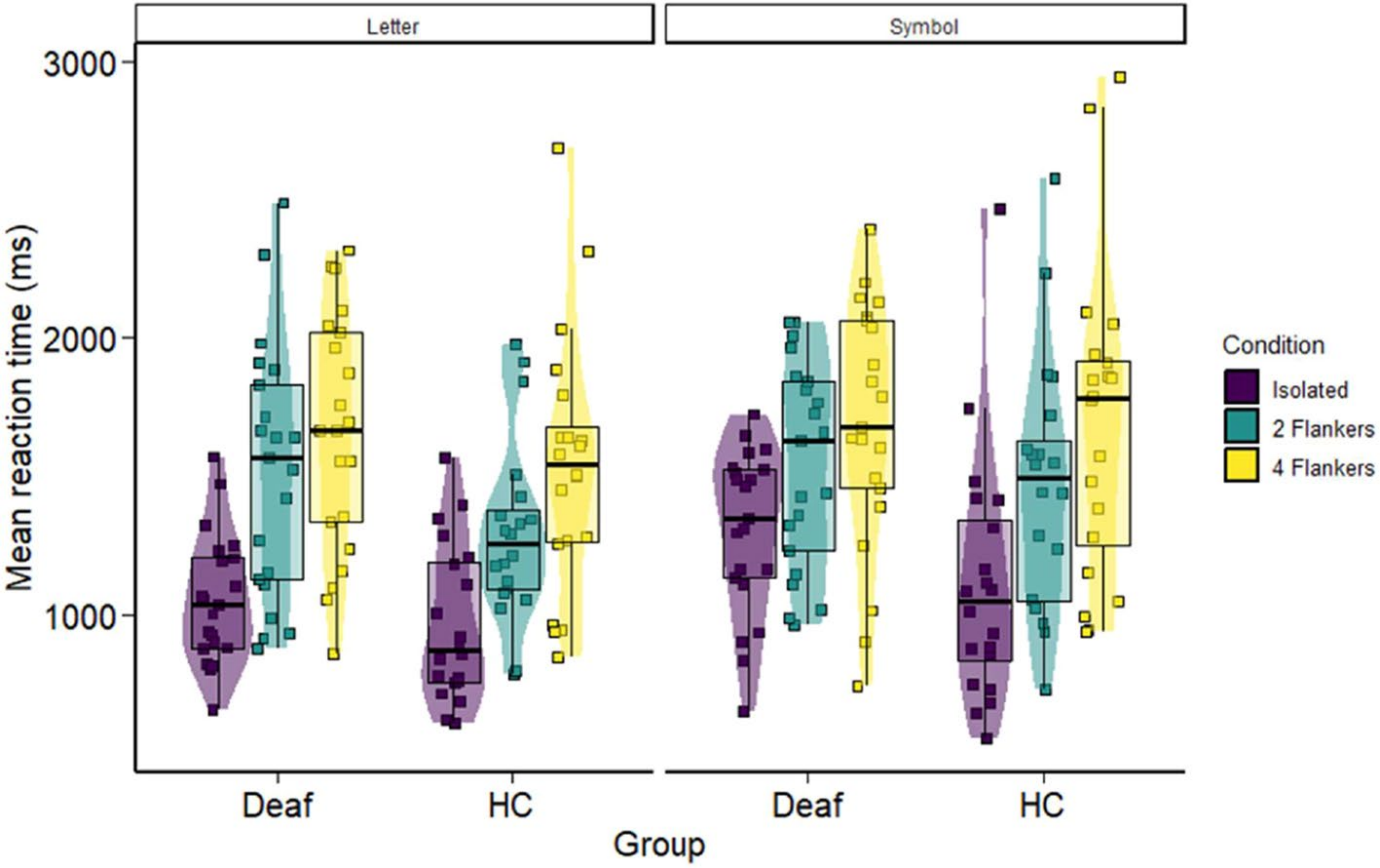
Violin plot depicting reaction times of deaf and hearing controls (HC) for letters and symbols in isolated (purple), 2 flankers (green), and 4 flankers (yellow) conditions.

A linear mixed model was fitted to predict RT with character, condition, side, and group (formula: RT ~ character * condition * side * group). The model included subjects as random effects (formula: ~ 1 | subject). Outliers with a standardized residual greater than 2.5 standard deviations were removed from the analyses (1.5% of the data)^39^. Results demonstrated a significant effect of character, *χ*^*2*^(*1*) = *39.84*, *p* < *0.01*, letters being recognized faster than symbols (*estimate* = *− 102*, *SE* = *12.9*, *t* = *− 7.90*, *p* < *0.01*). A condition effect was also highlighted, *χ*^*2*^(*2*) = *524.17*, *p* < *0.01*. Participants performed faster in the isolated condition than in the 2 flankers condition (*estimate* = *− 327*, *SE* = *14.8*, *t* = *− 22.15*, *p* < *0.01*). Performances in the 2 flankers condition was moreover faster than in the 4 flankers condition (*estimate* = *− 197*, *SE* = *16.6*, *z* = *− 11.9*, *p* < *0.01*). There was neither a side effect, *χ*^*2*^(*1*) = *0.63*, *p* > *0.4*, nor a group effect, *χ*^*2*^(*1*) = *1.31*, *p* > *0.2*. The results highlighted a character × condition × group interaction *χ*^*2*^(*2*) = *8.69*, *p* < *0.05*. This interaction revealed that the deaf group was faster for letters than symbols, but only when the letters were presented in isolation (*estimate* = *− 153.2*, *SE* = *29*, *t* = *− 5.29*, *p* < *0.01*), or surrounded by 2 flankers (*estimate* = *− 137.3*, *SE* = *31.1*, *t* = *− 4.42*, *p* < *0.01*). In contrast, no reaction time differences between letters and symbols appeared in the 4 flankers condition (*estimate* = *26.8*, *SE* = *35.5*, *t* = *0.75*, *p* > *0.9*). Unlike the deaf participants, the hearing group exhibited faster reaction times for letters compared to symbols under all conditions (isolation: *estimate* = *− 87.7*, *SE* = *27.4*, *t* = *− 3.20*, *p* < *0.05*; 2 flankers: *estimate* = *− 147.7*, *SE* = *30.6*, *t* = *− 4.82*, *p* < *0.01*; 4 flankers: *estimate* = *− 111.5*, *SE* = *34.9*, *t* = *− 3.19*, *p* < *0.05*) (see Fig. 3). No other effect was significant.

## Discussion

Several studies have shown that reading is particularly difficult for the deaf population, reading comprehension reaching a plateau at the fourth (9 years) or fifth (10 years) grade level^40^. While the literature examining the role language processes play in visual word recognition is becoming increasingly extensive, studies investigating the role of visual constraints at the letter recognition level are particularly scarce. The present study aimed to fill this gap by examining the visual crowding effect in a letter identification task. Deaf and hearing adults were asked to identify letters and symbols, presented individually or in meaningless sequences of 3 or 5 characters, to either the left or right of a fixation point.

Our results first revealed that letters were identified more accurately than symbols. Both deaf and hearing individuals therefore seem to develop a specialized letter-string system^14^. The presence of the crowding effect was moreover evidenced by the fact that characters (including both letters and symbols) were processed more efficiently when presented in isolation as opposed to when presented in a string of 3 or 5 characters. Deaf individuals processed both characters (letters and symbols) less accurately than their hearing peers when these characters were presented in isolation. They also exhibited less accurate letter identification performances compared to their hearing counterparts, although no significant differences were observed in symbol recognition. This observation is in line with previous studies suggesting that the deaf individuals’ reduced exposure to phonological information could impact letter processing^41–44^ much more than symbol identification, symbol identification being less reliant on phonological processing. However, as the phonological, and reading level of both groups was not assessed in our study, we cannot exclude the possibility that the observed difference merely reflects a difference in reading level or experience.

The response time data highlighted the same character and condition effects as the accuracy data but importantly revealed a significant character x condition x group interaction. In hearing, letters were processed faster than symbols in every condition. In deaf participants, target letters were processed faster than symbols when presented in isolation or with two flankers, but not when presented with four flankers. The allocation of spatial attention may therefore be influenced by the number of flanking elements^45^. Specifically, when flankers are adjacent to the target, such as in the two flankers condition, efficient deployment of spatial attention can suppress flanker interference. When flankers are not adjacent to the target, as in the four flankers condition, greater interference may in contrast occur due to inefficient spatial attention distribution over a longer string length. These results contrast with previous studies showing that deaf individuals outperform their hearing counterparts in detecting static or moving low-level visual stimuli in the periphery^46^ for a review, see^47^. They are however in line with a previous study investigating letter flanker interference effects in the deaf population^48^. In this last study, target letters were flanked by four similar or dissimilar letters at varying distances. While the hearing group exhibited faster reaction times at all distances, the deaf group demonstrated fewer errors but longer reaction times, particularly in the near periphery.

Deaf individuals may therefore develop a specialized system for letter identification, but its use may be more effortful than in hearing individuals, particularly at some eccentricities (in the near periphery). It is interesting to note that a line of text that falls on the retina can be segmented into three regions: the foveal region (1 degree of visual angle on each side of fixation, typically encompassing 6–8 letters in standard-sized print), the parafoveal region (extending up to about 5 degrees and accommodating 14–15 letters from the point of fixation), and the peripheral region (comprising everything beyond the parafovea)^1^. It would be interesting to examine whether deafness may shape letter discrimination abilities differently in these 3 regions.

Future studies should also investigate whether the potential slower peripheral reactivity to crowded letters could predict reading difficulty in deaf individuals. Finally, it would worth determining whether this difference between deaf and hearing people persists when reading level is controlled and when words are presented. Several models of word recognition (e.g., the Interactive Activation Model of visual word processing;^49^; OB-1 reader;^50,51^) indeed assume that meaningful strings of letters may strengthen or inhibit activations on the letter level. For example, seeing the word “WORK” would increase activation of the letters W, O, R, and K at the letter level and therefore resolve ambiguity and speeds up word recognition. In contrast, upon seeing “JUGDE”, the word-level representation for “JUDGE” would inhibit the letter G, allowing the correct letter D to be identified more clearly. When letter stimuli are briefly presented, letters in words are thus reported more accurately than letters embedded in non-words (i.e., “the word superiority effect”^52–54^). Top-down lexical-semantic representations therefore modulate letter-in-word processing. Since it has been assumed that skilled deaf reading may rely on precise orthographic representations and robust connections between orthography and semantics^30,55^, slow letter identification could be compensated for by the word superiority effect. As mentioned previously, deaf individuals experience differences in reactivity and redistribution of attention toward the periphery compared to hearing individuals in low-level visual tasks^47^. However, while exogenous (bottom-up processing) and endogenous (top-down processing) cues can facilitate the orientation of deaf adults’ visual attention, the exogenous facilitation effect was recently shown to be stronger for deaf participants than for hearing participants^56^. As the word superiority effect indexes top-down influences of lexical representations on letter identification, it will be interesting to examine whether the difference between exogenous and endogenous attention could also be observed in reading tasks.

To conclude, the present study demonstrates that deafness does not hinder the development of specialized letter processing that limits crowding. It nevertheless seems to slow down the process of identifying letter identities in strings of meaningless characters. Determining the role of visuospatial attentional abilities and the potential compensatory mechanisms at play during reading is crucial for a comprehensive understanding of reading processes in the deaf.

## Data Availability

Our data are freely available at the OSF link https://osf.io/bx8h3/.
